# CAR T cells as micropharmacies against solid cancers: Combining effector T-cell mediated cell death with vascular targeting in a one-step engineering process

**DOI:** 10.1038/s41417-023-00642-x

**Published:** 2023-06-30

**Authors:** Bianca Altvater, Sareetha Kailayangiri, Christian Spurny, Maike Flügge, Jutta Meltzer, Lea Greune, Katja Urban, Christian Schwöppe, Caroline Brand, Christoph Schliemann, Heike Hintelmann, Saliha Harrach, Wolfgang Hartmann, Hinrich Abken, Johannes Kuehle, Axel Schambach, Dennis Görlich, Wolfgang E. Berdel, Claudia Rossig

**Affiliations:** 1 Department of Pediatric Hematology and Oncology, University Children’s Hospital Muenster, Muenster, Germany; 2 Department of Medicine A, University of Muenster, Muenster, Germany; 3 Division of Translational Pathology, Gerhard-Domagk-Institute of Pathology, Münster University Hospital, Münster, Germany; 4 Division of Genetic Immunotherapy, Leibniz Institute for Immunotherapy (LIT), and University of Regensburg, Regensburg, Germany; 5 Center for Molecular Medicine Cologne, University of Cologne, 50931 Cologne, Germany; 6 Institute of Experimental Hematology, Hannover Medical School, Hannover, Germany; 7 REBIRTH Research Center for Translational Regenerative Medicine, Hannover Medical School, Hannover, Germany; 8 Division of Hematology/Oncology, Harvard Medical School, Boston Children’s Hospital, Boston, MA USA; 9 Institute of Biostatistics and Clinical Research, University of Muenster, Muenster, Germany; 10 Cells-in-Motion Cluster of Excellence (EXC 1003 - CiM), University of Muenster, Muenster, Germany; 11 Princess Máxima Center for Pediatric Oncology, Utrecht, The Netherlands

**Keywords:** Cancer immunotherapy, Drug development

## Abstract

To enhance the potency of chimeric antigen receptor (CAR) engineered T cells in solid cancers, we designed a novel cell-based combination strategy with an additional therapeutic mode of action. CAR T cells are used as micropharmacies to produce a targeted pro-coagulatory fusion protein, truncated tissue factor (tTF)-NGR, which exerts pro-coagulatory activity and hypoxia upon relocalization to the vascular endothelial cells that invade tumor tissues. Delivery by CAR T cells aimed to induce locoregional tumor vascular infarction for combined immune-mediated and hypoxic tumor cell death. Human T cells that were one-vector gene-modified to express a G_D2_-specific CAR along with CAR-inducible tTF-NGR exerted potent G_D2_-specific effector functions while secreting tTF-NGR that activates the extrinsic coagulation pathway in a strictly G_D2_-dependent manner. In murine models, the CAR T cells infiltrated G_D2_-positive tumor xenografts, secreted tTF-NGR into the tumor microenvironment and showed a trend towards superior therapeutic activity compared with control cells producing functionally inactive tTF-NGR. In vitro evidence supports a mechanism of hypoxia-mediated enhancement of T cell cytolytic activity. We conclude that combined CAR T cell targeting with an additional mechanism of antitumor action in a one-vector engineering strategy is a promising approach to be further developed for targeted treatment of solid cancers.

## Introduction

Adoptive cellular immunotherapy of cancer relies on the selective elimination of tumor cells by cytotoxic T cells or other immune effector cells. An advance in the field is the use of chimeric antigen receptors (CAR) to redirect the antigen specificity of T cells to tumor-associated surface antigens [1]. CAR T cells against the B lineage antigen CD19 have been highly effective to induce and even maintain remissions in patients with B cell-derived hematological malignancies [1]. A major goal in the field is to exploit the full potential of CAR T cell targeting also in solid tumors where some clinical responses have been achieved but remained incomplete and transient [2, 3]. T cell therapy of solid tumors is challenged by protective mechanisms of the tumor microenvironment (TME) (reviewed in [4]). Here we designed an innovative strategy that employs CAR T cells as micropharmacies to selectively deliver a therapeutic agent to the TME for a combination strategy that allows more potent control of solid tumors.

Our prototype CAR T cell micropharmacy employs an antivascular tumor targeting strategy based on induction of selective tumor vessel infarction by CD13-targeted truncated tissue factor (TF), a key initiator of the extrinsic coagulation cascade [5]. Truncating TF by removing the C-terminal transmembrane domain generates a soluble form (truncated TF, tTF) that lacks coagulation activity unless it is relocalized to the phospholipid membrane of a cell [5]. Small peptides containing the NGR motif (asparagine-glycine-arginine) bind to CD13 (aminopeptidase N) which is overexpressed on endothelial cells of proliferating blood vessels in both mice and humans, allowing NGR-coupled tTF to engage endothelial cells on the luminal side of tumor-associated vasculature with some level of tumor selectivity. In previous work, we have constructed fusion proteins consisting of NGR-peptide sequences coupled to the C-terminal end of tTF [6, 7]. tTF-NGR fusion proteins have thrombogenic activity in vitro, selectively bind to their target on endothelial cells (EC), and upon intravenous infusion in human xenograft mouse models induce thrombosis in tumor blood vessels with subsequent tumor growth retardation and regression [6, 7]. Within the therapeutically active dose range, no toxicity or thrombus formation was observed in histological preparations of healthy organs. In a phase I study, late-stage cancer patients treated with low, non-toxic doses tTF-NGR showed significant reduction of tumor blood flow in contrast to organ blood flow using contrast-enhanced ultrasound or magnetic resonance imaging [8].

To endow T cells with the ability to selectively produce and release the procoagulatory fusion protein tTF-NGR into the TME of human cancers, we adapted a previously described one-vector engineering strategy [9, 10]. A CAR directed against the surface ganglioside G_D2_ [11, 12] was retrovirally expressed in human T cells along with a CAR-inducible transgene encoding for recombinant tTF-NGR. G_D2_ is a target overexpressed on the cell surface of various cancers, including neuroblastoma and a proportion of bone sarcomas [13], breast [14] and lung cancers [15]. Engineered T cells secreted tTF-NGR with preserved procoagulatory function in a strictly CAR-antigen dependent manner, exerted robust effector cell responses against G_D2_-positive tumor targets and showed a consistent trend towards better antigen-specific antitumor activity in murine xenograft models compared to control CAR T cells secreting mutant non-functional tTF-NGR. Further technical improvements e.g. by allowing higher numbers of CAR T cells infiltrating the tumor and yielding higher amounts of secreted tTF-NGR payload may open promising avenues for double-targeted cellular therapy of solid tumors.

## Materials and methods

### General Lab Operation

The assays were performed by experienced individuals throughout the course of the study. The study was performed using established laboratory protocols covering the processing, freezing, storage and thawing of cells as well as the staining procedure, data acquisition and gating strategy. Additional data beyond the data found in the article are available from the corresponding author on reasonable request.

### Cell lines

A4573 were a gift from the Children’s Hospital Los Angeles, United States. HEK293T, Kelly, Jurkat, A204 and SUP-B15 were purchased from DSMZ (Braunschweig, Germany). Short tandem repeat (STR) profiling was used to confirm the identity of all cell lines. All cell lines were regularly checked by PCR to exclude mycoplasma contamination.

### Cell culture

Tumor cell lines were grown either as adherent cultures (HEK293T, A4573, Kelly and A204) or as suspension cultures (Jurkat and SUP-B15) on flasks in RPMI 1640 (Thermo Fisher, Dreieich, Germany) supplemented with 10% heat-inactivated fetal calf serum (FCS; Thermo Fisher, Germany) and 2 mM L-glutamine (Sigma-Aldrich, Taufkirchen, Germany), and maintained at 37 °C, 5% CO_2_ and high humidity.

### Constructs and transduction of human T cells

The cDNA coding for tTF, containing the extracellular amino acids GNGRAHA linked to the C-terminus (tTF-NGR), a histidine (HIS) tag and the human native tissue factor peptide leader coding sequence at the N-terminus, was designed either with a CMV promoter for constitutive expression or with an (NFAT)6mIL2 promoter for inducible expression and synthesized by Geneart (Thermo Fisher Scientific, Massachusetts, USA). The mutant tTF-NGR control variant (tTFmut-NGR) was designed with alanine substitutions of lysine residues 165 and 166 in TF. Both constructs were cloned into the retroviral vector pQCXIN [14] which contained an IRES site coupled GFP. For transduction of T cells, a retroviral one-vector gene pSERS11 [16]-based construct was used. The vector was cloned with an inducible (NFAT)6mIL2 promoter in the 5´ position and a constitutive PGK promoter in the 3’ position of the vector [17]. The cDNA for tTF-NGR and tTFmut_NGR was PCR cloned by Gibson Assembly (New England Biolabs, Frankfurt, Germany) into pSERS11 after the (NFAT)6mIL2 promoter in the 5´ position of the vector. The G_D2_-specific CAR GD2-BBζ was previously described [11, 18]. For production of retroviral supernatant, HEK293T cells were transfected together with the helper plasmids pEQPAM-3 and pRDF using K2® transfection reagent (Biontex, München, Germany, order no. T060). Supernatant was harvested on day 2 and day 3 after transfection and concentrated by centrifugation. Activation, transduction and expansion of T cells was performed as previously described [12].

### Western blot analysis

Transfected or transduced HEK293T or Jurkat cells were homogenized in 30–100 mL ice-cold radioimmunoprecipitation assay (RIPA) buffer (0.1% DTT, Sigma-Aldrich) with fresh protease inhibitor cocktail (Roche, Mannheim, Germany), shortly fractured in liquid nitrogen, thawed on ice, and then clarified by spinning for 15 min at 4 °C and 20 000 g. After measuring the protein concentration with Bradford reagent, 50 µg sample was separated by electrophoresis on an SDS 15% polyacrylamide gel and then electroblotted onto a nitrocellulose membrane (Bio-Rad, California, USA). Blocking was done in Tris-buffered saline with Tween® 20 (TBST) buffer containing 5% nonfat dry milk for 1 h, followed by incubation with rabbit anti-human TF IgG (Sekisui Diagnostics, Darmstadt, Germany, product no. ADG4502,) diluted 1:2 000 in TBST containing 5% BSA for 12 h at 4 °C. After washing, the membrane was incubated with horseradish peroxidase (HRP)-linked anti-rabbit immunoglobulin G (IgG) whole Ab (GE Healthcare, Munich, Germany, product no. NA934-1ML) at 1:3500 in TBST 5% nonfat dry milk for 2 h at RT, followed by washing and treatment with enhanced chemiluminescence reagent (ECL, Plus Western Blotting Detection System, GE Healthcare), and exposed to Hyperfilm ECL film (GE Healthcare) for 1 min. Equivalent protein loading was verified by Ponceau staining. To analyze deglycosylated tTF-NGR, 25 µg protein lysate was treated with 1 µl of Glycoprotein Denaturing Buffer (10X) in a total reaction volume of 10 µl, then heated at 100 °C for 10 min. After chilling on ice 2 µl GlycoBuffer 2 (10X), 2 µl 10% NP-40, 1 µl PNGase F (Promega, Walldorf, Germany, product no. V4831) and 6 µl H_2_O were added, mixed and incubated at 37 °C for 1 h.

### Factor X (FX) activation assay

The assay, originally established by Ruf et al. [19], is appropriate to describe the activation of the extrinsic coagulation cascade by TF and derivatives. In brief, 20 μL of the following was added to each well in a microtiterplate: (a) 50 nM recombinant FVIIa (Novo-Nordisc, Bagsværd, Denmark) in TBS containing 0.1% bovine serum albumin (BSA); (b) 25 − 250 pM tTF-NGR protein, in TBS-BSA; (c) 25 mM CaCl2 and 500 μM phospholipids (phosphatidylcholine/phosphatidylserine, 70/30, MM; Sigma, München, Germany). After 10 min at room temperature, the substrate FX (Enzyme Research Laboratories, Swansea, U.K.) was added in a final concentration of 1 μM. After additional 10 min, the reaction was stopped in 100 mM EDTA, and Spectrozyme FXa (American Diagnostica, Greenwich, USA; final concentration 0.7 mM) was added. The rates of FXa generation were monitored by the development of color at 405 nm with a microplate reader (Victor X3; PerkinElmer, Rodgau, Germany). Michaelis constants (K_D_) of the FX activation of tTF-NGR proteins were calculated by hyperbolic regression analysis as described by Hanes.

### Flow cytometry

CAR T cells were stained with fluorescence-conjugated monoclonal antibodies against CD3 (clone SK7, Biolegend, Heidelberg, Germany), CD8 (clone RPA-T8, Biolegend), CD4 (clone SK3, Biolegend), CD45RO (clone UCHL1, BD Pharmingen, Heidelberg, Germany), CD197 (clone G043H7, Biolegend), CD279 (clone EH12.2H7, Biolegend), TIM-3 (clone F38.2E2 eBioscience, Frankfurt, Germany), LAG-3 (clone 3DS223H, eBioscience) and the CAR-specific anti-idiotype antibody ganglidiomab, provided by H. Lode, Greifswald, Germany, and fluorescence-labeled with the Mix-n-Stain Kit (Sigma-Aldrich, Taufkirchen, Germany) or alternatively with a fluorescein isothiocyanate (FITC)-labeled goat-anti-human Fcγ Ab recognizing the hinge domain (Jackson ImmunoResearch, Pennsylvania, USA, product no. 109-095-098) for the CD19CAR T cells. Samples were fixed with 1% paraformaldehyde (PFA) and acquired directly or not later than 24 h after staining. For each sample, 10 000 cells within the respective gates were analyzed with FACS Diva 9.0, using FACS Celesta flow cytometer (BD Biosciences, Heidelberg, Germany) and FlowJo version 10 (FlowJo, LLC, Ashland, OR, USA).

### Intracellular staining

T cells (5 × 10^5^) 13–14 days after transduction and target cells (5 × 10^5^) were coincubated for 2 h at 37 °C and 5% CO_2_. 10 µg of Brefeldin A in 40 µl PBS (Sigma Aldrich, Germany) was added for 4 h to block cytokine secretion. After washing with PBS supplemented with 0.5 % BSA, the CF488-conjugated anti-idiotype antibody ganglidiomab and CD3-specific antibody were added and cells were incubated for 15 min at RT. Following another washing step, cells were incubated with FACS Lysing Solution diluted 1:10 in dH_2_O (BD, Biosciences, Germany) for 10 min at RT. Then the cells were washed and treated for another 10 min at RT with FACS Permeabilizing Solution (Becton Dickinson, Heidelberg, Germany), again diluted 1:10 in dH_2_O. After a washing step, cells were resuspended in 100 µl washing buffer containing antibodies against IFN-γ (clone B27, BD) and TNF-α (clone mAb11, Biolegend) and incubated for 30 min at RT. After a final washing step, cells were fixed with 1% PFA and analyzed by flow cytometry. T cells incubated with medium alone served as controls. For Granzyme B analysis, the CAR T cells were either incubated with vehicle (H_2_O) or 50 µM DMOG for 72 h and then prepared as above for intracellular staining, followed by incubation with the GranzymeB antibody (clone QA18A28, Biolegend).

### Cytotoxicity assay

Tumor cytolysis was assessed in a calcein-AM release assay, as previously described in detail [12]. In brief, target cells at a final concentration of 2 × 10^6^ cells/ml were incubated with calcein-AM at 10 μM (Thermo Fisher, Germany) for 30 min at 37 °C, then coincubated in triplicates in flat bottom 96-well microtiter plates (Thermo Scientific, Germany) with CAR T cells at effector-to-target (E:T) cell ratios from 12:1 to 6:1. Additional triplicate wells were set up to assess spontaneous (target cells alone in complete medium) and maximum release (target cells in medium plus 9% Triton X-100). After 4 h at 37 °C in 5% CO_2_, samples were transferred to black-walled 96-well microtiter plates (Greiner Bio-One, Solingen, Germany) and measured using the GloMax® Discover multi-mode microplate reader (Promega, Germany) at excitation 475 nm and emission 500–550 nm. Data were expressed as arbitrary fluorescent units (AFU). Specific lysis was calculated by using the formula [(test release - spontaneous release)/(maximum release - spontaneous release)] × 100. For analysis of hypoxia, the CAR T cells were either incubated with vehicle (H_2_O) or 50 µM DMOG for 72 h prior to the assay.

### CD107a assay

Target cells and effector cells were treated with 1 µL/ml Monensin (Biolegend) and co-cultured at a 1:1 ratio for 3 h at 37 °C, in the presence of CD107a BV510 (clone H4A3 BD Horizon). Then, the cells were washed and stained with CF488 fluorescence-labeled CAR anti-idiotype antibody ganglidiomab and APC-Fire750-labeled anti-CD3 (clone SK7, Biolegend) for 15 min in the dark before analysis by flow cytometry.

### ELISA

CAR T cells from two individual donors were continuously stimulated with anti-CD3/CD28 antibodies (1 µg/ml each) or anti-idiotype antibody ganglidiomab (1 µg/ml) or by coincubation with G_D2_-pos, CD19-neg tumor cells (A4573, Kelly) or G_D2_-neg, CD19-pos leukemia cells (SUP-B15). Medium alone or the G_D2_-neg, CD19-neg cell line A204 was used as a control. Supernatant was harvest after 48 h. For analysis of release kinetics of tTF-NGR and tTFmut-NGR the supernatant was harvested at the indicated points in time after continuous stimulation with anti-CD3/CD28 antibodies (1 µg/ml each) with two medium changes after 24 and 48 h. ELISA was performed with the simple step tissue factor ELISA as recommended by the manufacturer (ab220653 Abcam, Hamburg, Germany). For analysis of tTF-NGR and tTFmut-NGR released into the tumor tissue, a section of the tumor was cryofixed and lysed using Cell Extraction Buffer PTR provided with the kit. The ELISA has a sensitivity of 3.6 pg/ml and a detection range of 15.63 to 1000 pg/ml.

### Immunohistochemistry

Formalin-fixed paraffin-embedded sections of human tumor biopsies or xenografts were deparaffinized and either stained with human CD31-specific antibody (clone JC70, Cell Marque, California, USA) or a human CD13-specific antibody (clone SP10087, Cell Marque, USA) using Bench Mark Ultrastainer (Ventana, Roche, Basel, Switzerland). Imaging was perfomed using Vectra® 3.0 system (Perkin Elmer, Germany) and InForm Analysis Software. For H&E staining, formalin-fixed paraffin-embedded sections of xenografts were stained with hematoxylin (Merck, Darmstadt, Germany) for 3 min and eosin (Roth, Karlsruhe, Germany) for 2 min. The tissue sections were mounted with Vitrocloud (Langenbrink, Bissendorf, Germany). Imaging was perfomed using Vectra® 3.0 system (Perkin Elmer, Germany) and InForm Analysis Software. For EVG staining, Formalin-fixed paraffin-embedded sections of xenografts were stained with resorcinol fuchsin (Waldeck, Germany), picrofuchsin (Waldeck, Germany) and hematoxylin according to manufacturer´s instruction using the Tissue Tek Prisma^®^ (Sakura, Düsseldorf, Germany) autostainer. Imaging was performed using Vectra® 3.0 system (Perkin Elmer, Germany) and InForm Analysis Software.

### Multiplex fluorescence staining

Multiplex fluorescence staining was perfomed by the tyramide signal amplification (TSA) method using Opal fluorophores (Akoya Biosciences, Marlborough, USA). Microwave treatment between the staining cycle was used to remove primary and secondary antibodies, while retaining the fluorescent signal. Formalin-fixed paraffin-embedded sections of xenografts were deparaffinized, rehydrated and washed in water before staining with two staining cycles. First staining cycle: antigen retrieval (AR) with citrate buffer pH 9, blocking with normal goat serum (NGS) for 1 hr at RT, incubation with anti-Granzyme B antibody (clone D6E9W, Cell Signaling, Massachusetts, USA), diluted 1:100 in TBST supplemented with 5% NGS overnight at 4 °C, incubation with secondary antibody (polymer HRP Ms+Rb, ARH1001EA, Akoya Bioscience) for 10 min at RT, then incubation with Opal 570 (1:100) (FP1488001KT, Akoya Bioscience) for 10 min at RT. Second staining cycle: microwave treatment with citrate buffer pH 6, blocking with serum-free blocking solution (Dako) for 1 h at RT, incubation with anti-CD8 antibody (clone SP57, Ventana, Basel, Switzerland) (undiluted) for 30 min at RT, incubation with secondary antibody as above, then incubation with Opal 690 (1:150) (FP1497001KT, Akoya Bioscience) for 10 min at RT. Then the slides were counterstained with DAPI (Biocat, Heidelberg, Germany) and enclosed with Prolong Diamond Antifade (P36961,Thermo Fisher). Imaging was performed with Vectra® 3.0 system (Perkin Elmer, Germany). A whole slide scan was first acquired at 10x magnification, across the full emission light spectrum in each filter tube: DAPI (450–470 nm), FITC (505–545 nm), CY3 (580–620 nm), Texas Red (600–650 nm), and CY5 (670–720 nm). Five regions of each slide were chosen for multispectral imaging, using the image analysis software Phenochart™, and were re-scanned at 20 × magnification. Multispectral images were unmixed using spectral libraries built from images of single-stained tonsil for each individual fluorophore, with the InForm Analysis Software (InForm 2.1.4, Akoya Bioscience). The autofluorescence was subtracted from unmixed multispectral images of an EwS tissue slide that had undergone the complete staining procedure, omitting the primary antibodies. The images were trained using the InForm software (tissue segmentation, cell segmentation, and phenotyping tools). The same algorithms were used to analyze granzyme B + /CD8+ cells within the xenograft tissues. Data processing and analysis were performed with R software version 3.6.3 and integrated development environment RStudio version 1.4., with packages phenoptrR and phenoptr Reports (Akoya Bioscience).

### RNA in situ hybridization (RNAish)

RNAish was performed on xenografts using Hs-Hif1A probe (605221, ACD Bio, Newark, USA), an RNAscope® Multiplex Fluorescent Reagent Kit v2 (323100, ACD Bio, USA) and the Opal fluorophores, according to the manufacturer’s instruction. Briefly, formalin-fixed paraffin-embedded xenografts were deparaffinized and treated with hydrogen peroxidase prior antigen retrieval in a pressure cooker. Then slides were treated with protease, followed by hybridization and signal amplification. Next, slides were blocked with TBST supplemented with 1 % BSA and 10% normal goat serum overnight at 4 °C, followed by incubation of anti-CD8 antibody (clone SP57, Ventana, Switzerland) for 1 h at RT, incubation with the secondary antibody (polymer HRP Ms+Rb) for 10 min and Opal 690 (1:1000) for 10 min at RT. All slides were counterstained with DAPI (Biocat, Germany) and enclosed with Prolong Diamond Antifade (Thermo Fisher). Imaging was performed with Vectra® 3.0 system (Perkin Elmer, Germany). Three regions containing T cells of each slide were randomly chosen for multispectral imaging and were unmixed using spectral libraries with the InForm Analysis software. The images were trained using the InForm software (tissue segmentation, cell segmentation, and phenotyping tools). The same algorithms were used to analyze Hif1 alpha +/CD8+ cells within the xenograft tissues. Data processing and analysis were performed as described above for multiplex fluorescence staining.

### In vivo experiments

Mouse experiments were approved by the animal care committee of the local government (LANUV, Recklinghausen, Az. 84-02.04.2015.A227). NSG mice were originally purchased from Charles River (Sulzfeld, Germany) and used from own breeding in the central animal experimental facility Muenster (ZTE). Animals were housed in pathogen-free rooms in type-2L individually ventilated cages (Charles River) with a maximum of 6 mice per cage, with access to sterile food and water ad libitum and a constant RT at 21 °C. NSG mice of both genders and 8–12 weeks old were used for the experiments. Sex of animals was distributed equally between cohorts, if applicable. Sample size were chosen by using the Resource Equation method described by Festing et al. [20]. Mice were randomized per cage and no blinding was performed. To establish tumor xenografts, 5 × 10^6^ cells of the NBL cell line Kelly or EwS cell line A4573, respectively, were injected s.c. into the right flanks of NSG mice. Tumor growth was monitored by caliper measurements. Tumors after sacrifice were immediately dissociated in PBS and singularized by passage through a cell strainer, followed by incubation with Red blood lysis buffer (Qiagen, Hilden, Germany) and flow cytometry. Mice were excluded from analysis if death was not related to tumor progression or therapy.

### Patient material

Immunohistochemical analyses were performed on pretherapeutic tumor biopsies from patients with EwS treated at University Children´s Hospital, Muenster, within the multicenter clinical studies EURO-E.W.I.N.G. 99 and EWING 2008 (EudraCT 2008-003658-13) approved by the Institutional Review Board (Az. 2008-391-f-A). Patients and/or their legal guardians had consented to the use of biopsy material for research purposes in accordance with the Declaration of Helsinki.

### Statistical analysis

Data were analyzed and visualized using IBM SPSS Statistics 29 for Windows and Systat SigmaPlot 11.0 software. Continuous variables are described by mean and standard deviation. Categorical variables are described by absolute and relative frequencies. Statistical testing was performed using parametric and non-parametric methods (ANOVA on ranks). Details are given in figure captions, respectively. Results were considered to be statistically significant at p ≤ 0.05. Overall survival time of mice was defined from start of therapy until death of any cause or completion of experiment on day 42. Mice alive with tumor or in CR were censored at the end of the observation period. The Kaplan-Meier method [21] was used to analyze overall survival rates.

## Results

### Eukaryotic cells including human T cells can produce functionally active tTF-NGR

In our previous preclinical work and for use in humans in clinical studies, we produced tTF-NGR in prokaryotic cells [6–8]. As a first step toward CAR T cell-induced secretion of tTF-NGR, we investigated whether the fusion protein is effectively produced and functionally assembled also by human cells. To generate both constitutive and CAR-inducible expression cassettes encoding for tTF-NGR, the cDNA coding for tTF containing the extracellular amino acids GNGRAHA, in which the heptapeptide is linked to the C-terminus of tTF (tTF-NGR), was cloned into the pQCXIN vector [10]. The gene product also has an N-terminal histidine (HIS) tag for purification of the protein by using immobilized metal-chelate affinity chromatography. The human TF peptide leader coding sequence [22] was cloned N-terminal for optimal protein secretion. Constitutive gene expression is achieved under control of the CMV promoter whereas in the inducible construct tTF-NGR release is driven by the (NFAT)_6_mIL2 promoter consisting of six nuclear factor of activated T cells (NFAT) response elements, NFAT_6_, fused to a minimal IL-2 promoter, to allow selective induction by the downstream events of CAR T cell ζ signaling [23]. To control for tTF-NGR function, we generated a mutant tTF-NGR control variant (tTFmut-NGR) inadequate to induce coagulation, by alanine substitutions of lysine residues 165 and 166 in TF [24].

**Fig. 1:**
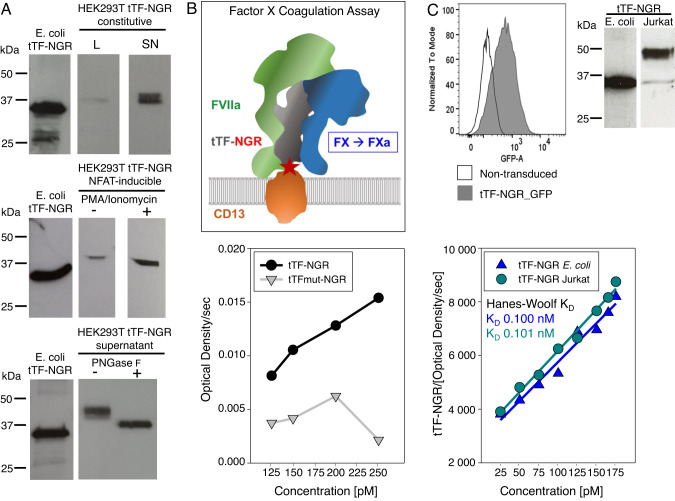
Human T cells can produce functionally active tTF-NGR protein. **A** Western Blot detecting tTF-NGR protein in lysates (L) and supernatants (SN) of transiently transfected HEK293T cells using a constitutive expression vector (upper panel) or NFAT-inducible expression vector (middle panel), with (+) and without (-) stimulation with PMA (25 ng/ml) and ionomycin (1 µg/ml) for 24 h at 37 °C. Lower panel: Molecular weights of tTF-NGR by Western Blot in the supernatants of transfected HEK293T cells treated with peptide-N-glycosidase F (PNGase F) at 500 U/25 µg protein for 1 h at 37 °C (+, treated; -, untreated) compared to control tTF-NGR protein produced by competent *E. coli* strain BL21 DE3. **B** Upper panel: Schematic visualization of the Factor X activation assay to assess the ability of tTF-NGR in presence of a phospholipid-rich milieu (here cell membrane) within the complex tTF-NGR:FVIIa:FX to activate FX to FXa as a pro-coagulatory step. Lower panel: Michaelis-Menten plot representing the capacity of tTF-NGR versus mutated tTF-mutNGR from supernatants of transfected HEK293 cells to enhance the specific activation of factor FX to FXa in a dose-dependent fashion. **C** Upper left panel: Jurkat cells were transduced to express the tTF-NGR expression cassette by retroviral gene transfer, and the transduction efficiency was quantified by flow cytometry using the GFP marker gene. Upper right panel: Western Blot showing tTF-NGR protein produced by Jurkat cells in comparison to *E.coli*. Lower panel: Hanes-Woolf plot showing the equivalent capacity of tTF-NGR from supernatants of transduced Jurkat cells compared to the standard recombinant tTF-NGR protein produced by *E. coli* BL21 to enhance the proteolytic activation of FXa in a Factor X activation assay. Representative experiment of 3.

Transfection of human HEK293T cells resulted in tTF-NGR protein detectable by Western blot analysis in supernatants (SN) and at low concentrations also in lysates of the transfected cells (Fig. 1A). When using the inducible expression system, the amount of detected protein was enhanced by stimulation of the cells with phorbol myristate acetate (PMA) and ionomycin which activate NFAT. Compared to *E. coli* protein, tTF-NGR expressed by human cells was found to have a larger size. Enzymatic treatment with the endoglycosidase peptide:N-glycosidase F (PNGase F) substantially reduced the size, demonstrating that the difference in size is due to glycosylation. To investigate whether tTF-NGR produced by human cells retains its procoagulatory activity, we performed a coagulation assay that describes activation of the extrinsic coagulation cascade by TF [19]. The ability of the fusion protein to enhance the specific proteolytic activation of factor (F) X by complex-building with FVIIa is quantified by Michaelis–Menten analysis. tTF-NGR from the supernatants of transfected cells is functionally active since it effectively enhanced FX activation, in contrast to supernatants from non-transfected HEK293T cells and from cells transfected with the mutant tTF control variant (Fig. 1B).

Next we investigated the capacity of human T cells to produce functional tTF-NGR. By retroviral gene transfer of tTF-NGR along with GFP expressed downstream of an internal ribosomal entry site (IRES) as marker for detection, we achieved effective transduction of the human T cell line Jurkat with the tTF-NGR transgene (Fig. 1C, upper left panel). Western Blot analysis confirmed that the transduced Jurkat cells produce tTF-NGR protein (Fig. 1C, upper right panel). Again, tTF-NGR produced by Jurkat cells compared to *E. coli* had a higher molecular weight, reflecting glycosylation in eukaryotic cells. From 150 ml supernatant of transduced Jurkat cells, we extracted approximately 1 μg of tTF-NGR protein for functionality analysis. Quantification of FX activation revealed similar Michaelis constants (K_D_) for tTF-NGR produced by *E. coli* (0.124 ± 0.014 nM, n = 3) and the glycosylated human variant produced by human T cells (Fig. 1C, lower panel). Thus, the protein modifications by human T cells do not interfere with the ability of recombinant tTF-NGR to enhance FX activation and thus retain its procoagulatory activity.

### Human T cells transduced to express genes encoding for a G_D2_-specific CAR and CAR-inducible tTF-NGR functionally interact with G_D2_-positive tumor targets

**Fig. 2:**
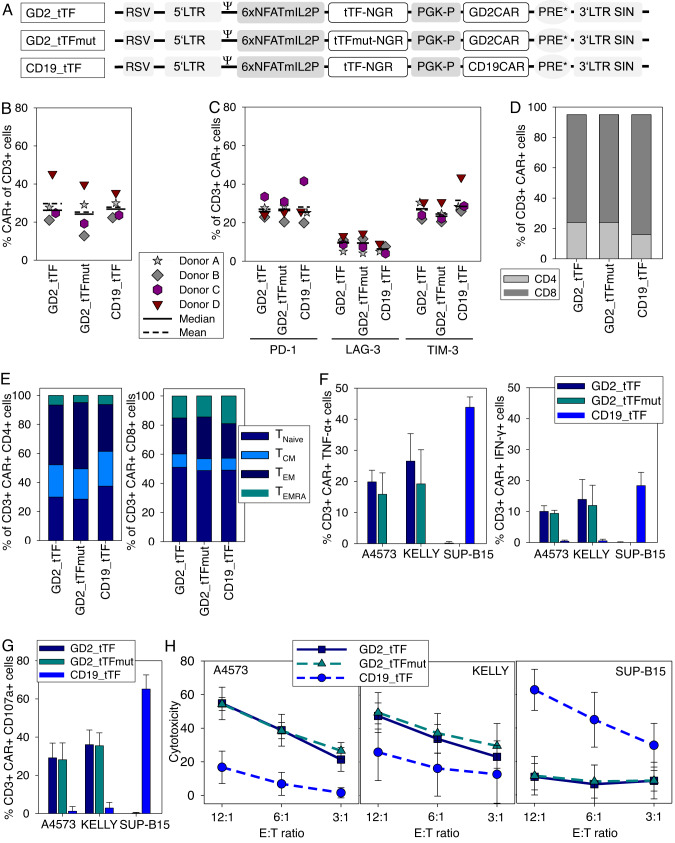
Human T cells transduced to express genes encoding for a G_D2_-specific CAR and a CAR-inducible tTF-NGR functionally interact with G_D2_-positive tumor targets in an antigen-specific manner. **A** Design of all-in-one retroviral vectors for CAR-inducible production of tTF-NGR or tTFmut-NGR as control. **B** Detection of CAR surface expression in transduced T cells from 4 human donors on day 14 after transduction by flow cytometry detection of the scFv component of the CAR using anti-idiotype antibody ganglidiomab (GD2_tTF, GD2_tTFmut) or anti-Fc-γ (CD19_tTF), respectively. **C** Surface expression of exhaustion markers PD-1, LAG-3 and TIM-3 on transduced T cells. **D** CD4 and CD8 ratios and **E** proportions of CD3 + T cells with naive (T_Naive_), central memory (T_CM_), effector memory (T_EM_) and CD45RA+ effector memory (T_EMRA_) phenotypes, all assessed on day 14 after transduction by flow cytometry. **F** Secretion of TNF-α and IFN-γ by T cells transduced with the indicated constructs in response to coincubation with G_D2_-pos, CD19-neg tumor cells (EwS cell line A4573, NBL cell line Kelly) or G_D2_-neg, CD19-pos leukemia cells (SUP-B15) by flow cytometry. **G** CD107a release by T cells transduced with the indicated constructs in response to coincubation with G_D2_-pos, CD19-neg tumor cells (A4573, Kelly) or G_D2_-neg, CD19-pos leukemia cells (SUP-B15). **H** Cytolysis of G_D2_-pos, CD19-neg tumor cells (A4573, Kelly) or G_D2_-neg, CD19-pos leukemia cells by T cells transduced with the indicated constructs. All analyses were done with 4 human donors and either shown as individual data or as means with standard deviations.

To investigate the capacity of a tumor-antigen specific CAR to induce the release of tTF-NGR in response to tumor cells, we coexpressed both genes in primary human T cells. The CAR targets ganglioside G_D2_ and employs 4-1BB for costimulation [25, 26]. To minimize manipulation of the T cells and ensure optimal in vivo function, we used a novel one-vector retroviral gene transfer system. The inducible protein expression cassette was designed in the 5’ position, and the constitutive CAR cassette in the 3’ position. This architecture allows independent expression of a CAR-induced transgene driven by the CD3ζ-activated (NFAT)_6_mIL2 promoter. On the retroviral vector backbone pSERS11 [16, 27] we generated vectors encoding for constitutive expression of the G_D2_-specific CAR and inducible tTF-NGR (GD2_tTF) or mutant tTF-NGR control variant (GD2_tTFmut), and a control targeting the B-lineage antigen CD19 along with inducible tTF-NGR (CD19_tTF) (Fig. 2A).

Transduction of T cells from 4 healthy donors resulted in CAR surface expression in a median of 26% (range 21 to 45 %) transduced with GD2_tTF, 24% (range 13 to 40%) with GD2_tTFmut and 27% (range 22 to 35%) with CD19_tTF (Fig. 2B). T cells transduced with either the G_D2_-specific or the CD19-specific control CAR along with tTF-NGR (or tTFmut-NGR) had comparable proportions of T cells expressing the activation/exhaustion markers PD-1, LAG-3 and TIM-3 (Suppl. Fig. 1, Fig. 2C) and comparable CD4 to CD8 T cell ratios (Fig. 2D) and T cell differentiation phenotypes (Fig. 2E). They responded to stimulation by secretion of TNF-α and IFN-γ (Fig. 2F) and by upregulation of the T cell degranulation marker CD107a (Fig. 2G) in a target-specific manner. Moreover, transduced T cells effectively lysed G_D2_-positive or CD19 expressing tumor target cells, respectively, in the absence of background cytolysis of antigen-negative cells (Fig. 2H). Overall, G_D2_ antigen engagement on tumor cells by T cells engineered with a G_D2_-specific CAR and CAR-inducible tTF-NGR induces a potent and target-specific T cell activation and effector response.

### Human T cells coexpressing a G_D2_-specific CAR and CAR-inducible tTF-NGR secrete tTF-NGR in an antigen-inducible manner

**Fig. 3:**
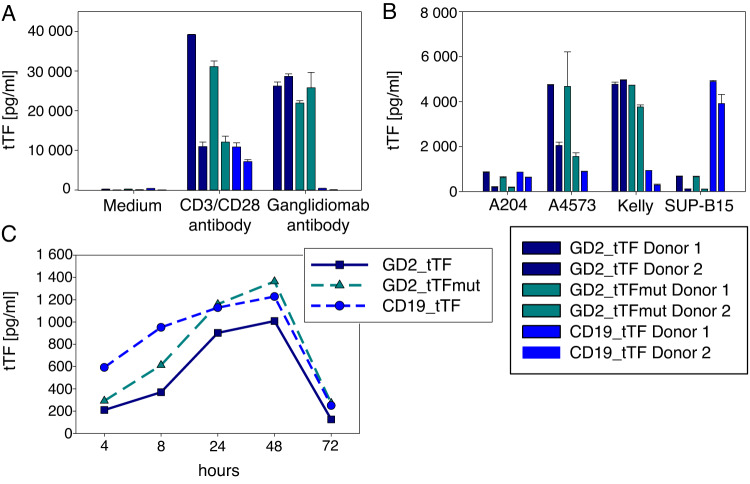
Human T cells transduced to express GD2_tTF, GD2_tTFmut or CD19_tTF produce recombinant tTF-NGR or tTFmut-NGR in an antigen-inducible, strictly antigen-specific manner. **A** Concentrations of tTF-NGR and tTFmut-NGR quantified by ELISA in the supernatants of human T cells from two individual donors transduced with the indicated constructs and stimulated on day 14 after transduction with anti-CD3/CD28 antibodies (1 µg/ml each) or with anti-idiotype antibody ganglidiomab (1 µg/ml) or with medium alone (negative control), or (**B**) by coincubation with G_D2_-pos, CD19-neg tumor cells (A4573, Kelly) or G_D2_-neg, CD19-pos leukemia cells (SUP-B15), or as negative control with the G_D2_-neg, CD19-neg cell line A204 (**C**) Kinetics of the concentrations of tTF-NGR and tTFmut-NGR by ELISA in the supernatants of transduced T cells at the indicated times after continuous stimulation with CD3 antibody, with two medium changes after 24 and 48 h.

Antigen-specific stimulation of GD2_tTF- and GD2_tTFmut-expressing T cells using anti-idiotype antibody ganglidiomab (Fig. 3A) or by coincubation with G_D2_-positive tumor targets (A4573, Kelly) (Fig. 3B) induced secretion of tTF-NGR or tTFmut-NGR, respectively. Production of tTF-NGR (or control tTFmut-NGR) was also induced by stimulation via the native TCR by antibodies against CD3 and CD28, as expected (Fig. 3A). Remarkably, the T cells did not produce tTF-NGR or tTFmut-NGR in the absence of stimulation (medium control, Fig. 3A) or in the presence of G_D2_-negative target cells (A204, Fig. 3B), nor in response to target cells expressing CD19 but not G_D2_ (Sup-B15, Fig. 3B). In comparison, tTF_CD19 CAR-transduced T cells secreted the fusion protein in response to CD19-positive but not G_D2_-positive target cells. Thus, production of the recombinant coagulation factor strictly relies on signals provided by TCRζ or CAR signaling.

To study the kinetics of NFAT-induced, antigen-induced protein release, we subjected the transduced T cells to a single round of stimulation with anti-CD3 antibody, followed by quantification of the fusion proteins in the cell supernatants. tTF-NGR and tTFmut-NGR concentrations continued to increase until a maximum concentration was reached at 48 h, returning to baseline after further 24 h (Fig. 3C). Since continued T cell culture beyond 24 h after stimulation requires medium changes, the peak concentrations at 48 and 72 h reflect only freshly released tTF-NGR after 24 and 48 h, respectively. We conclude that dual-engineered T cells can act as micropharmacies that produce and release functional tTF-NGR in response to tumor targets in a CAR-inducible, strictly antigen-specific manner.

### GD2_tTF gene-modified T cells can infiltrate human neuroblastoma tumor xenografts and release tTF-NGR into the TME without systemic toxicities

**Fig. 4:**
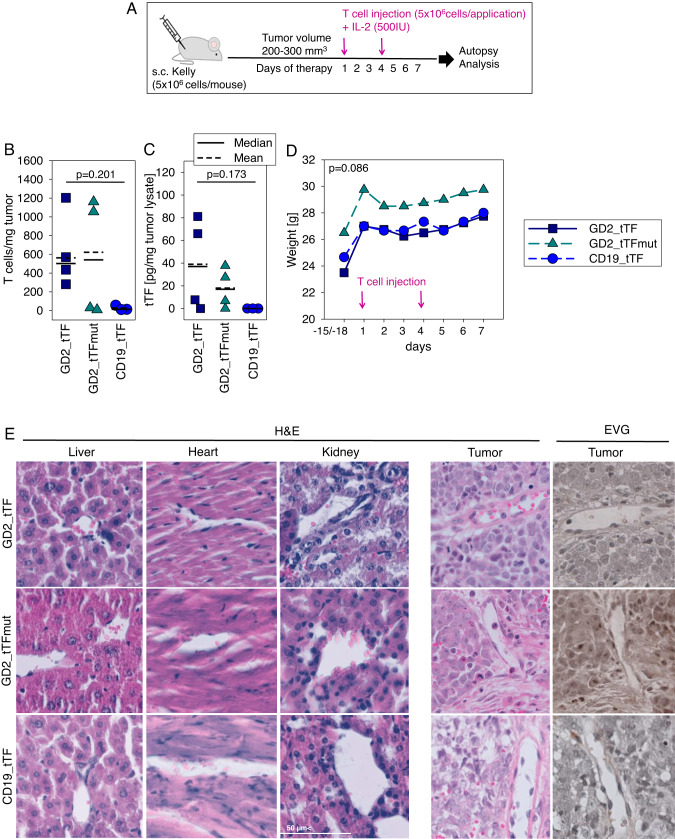
In vivo proof of concept experiment addressing tumor infiltration and local release of tTF-NGR by GD2_tTF gene-modified T cells along with local and systemic effects of T cell administration in a murine neuroblastoma xenograft model. **A** Schematic illustration of the experimental design. **B** Numbers of human CD3 + T cells in dissociated tumor xenografts by flow cytometry on day 7. **C** Amounts of tTF-NGR or tTFmut-NGR in tumor lysates by ELISA on day 7. **D** Weight monitoring of mice during and after administration of CAR T cells. **E** H&E staining (murine liver, heart, kidney and tumor) and Elastika van Gieson (EVG) staining (tumor) of paraffin-embedded tissue sections on day 7 after administration of CAR T cells. All analyses were done with 4 mice per cohort (GD2_tTF, GD2_tTFmut) or 3 mice (CD19_tTF) and either shown as individual data or as means and significance was assessed using ANOVA on Ranks.

In the first in vivo experiment, we aimed to demonstrate the ability of the transduced T cells to deliver tTF-NGR into the TME of human tumor xenografts. We used a localized neuroblastoma model based on subcutaneous (s.c.) growth of the G_D2_-positive cell line Kelly. Once tumor volumes reached 200–300 mm^3^, human T cells transduced with GD2_tTF, GD2_tTFmut or CD19_tTF were intraperitoneally (i.p.) administered on days 1 and 4, along with human interleukin-2 (Fig. 4A). T cell numbers as indicated reflect numbers of transduced (CAR-expressing) cells. The i.p. route of CAR T cell administration had previously been found to be equally effective against s.c. tumors as intratumoral or intravenous delivery [28]. The experiment was stopped on day 7 to analyze parameters of action of tTF-NGR secreting CAR T cells. Mice were sacrificed and tumors and organs removed for histological and flow cytometry analysis. Anti-human CD3 staining of dissolved tumor tissue and flow cytometry analysis revealed the presence of CAR T cells in the majority of tumor tissues from mice receiving G_D2_-targeted CAR T cells (Fig. 4B). tTF-NGR or tTFmut-NGR were detected in tumor lysates from mice treated with respective GD2-specific CAR T cells by ELISA, though not in all, and at different concentrations among individual animals (Fig. 4C). Neither T cells nor tTF-NGR was detected in tumors from mice receiving CD19-targeted T cells (CD19_tTF).

Systemic administration of tTF-NGR up to doses of 3 mg/m^2^ is safe in humans [8]. Still, background release of high amounts of tTF-NGR into the systemic vasculature could potentially cause significant thromboembolic side effects. Monitoring of the weights of the animals did not reveal any signs of systemic toxicity after administration of CAR T cells containing tTF-NGR (Fig. 4D). Moreover, H&E staining of paraffin-embedded tissue sections from murine organs (liver, kidney and heart) did not demonstrate vascular infarction or tissue injury (Fig. 4E). Thus, we show that G_D2_-specific CAR T cells with inducible tTF-NGR can localize to the microenvironment of G_D2_+ neuroblastoma xenografts and locally deposit tTF-NGR. Next, we addressed whether this results in locoregional coagulation, the postulated mechanism of tTF-NGR action. Against our hypothesis, analysis of tumors on day 7 after CAR T cell administration failed to detect microscopically visible presence of blood pooling, vascular disruption or other signs of tumor vascular infarction in the targeted tumor tissue (Fig. 4E), possibly due to the small amounts of tTF-NGR secreted into the tumor.

### GD2_tTF gene-modified T cells show a trend towards superior antitumor activity against Ewing sarcoma xenografts compared to control CAR T cells without coagulatory activity

To investigate the combined activity of G_D2_-specific CAR T cell effector responses with antigen-inducible release of tTF-NGR, we used a murine xenograft model of Ewing sarcoma (EwS). EwS is a highly vascularized tumor that expresses the NGR-target CD13 on tumor vascular endothelial cells, confirmed by immunohistochemistry analysis of CD31 and CD13 in 6 human tumor biopsies (Suppl. Fig. 2) [29]. EwS often expresses G_D2_ [12] and has a solid tumor architecture not usually infiltrated with T cells and relatively resistant to CAR targeting in vivo [30].

**Fig. 5:**
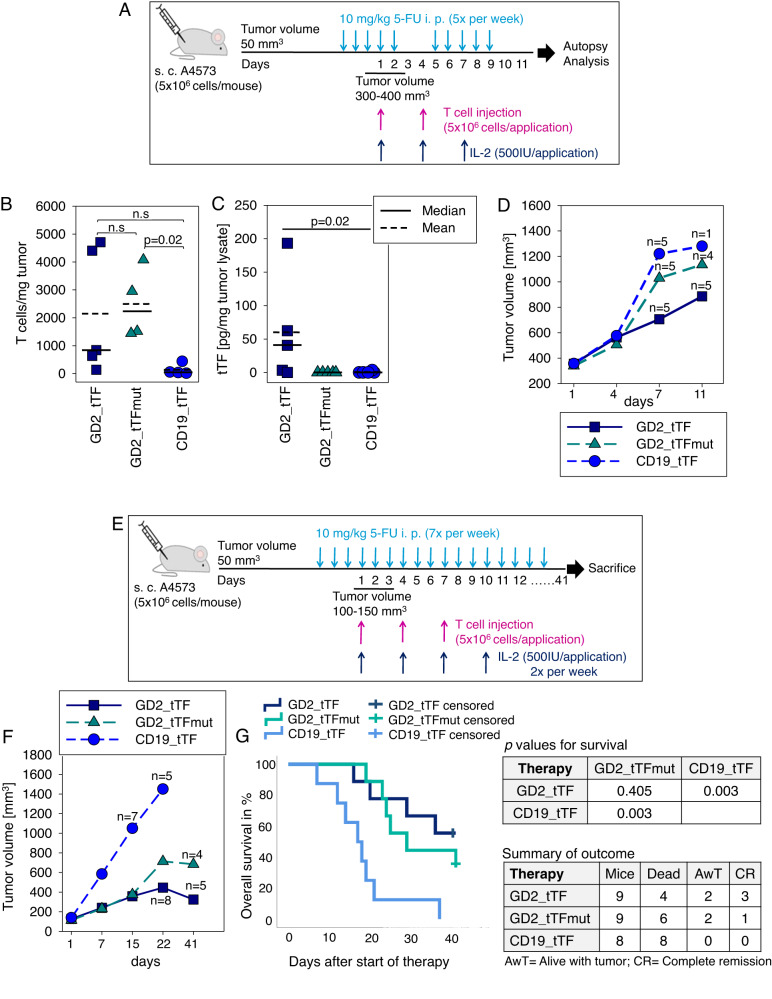
Anti-tumor activity of gene-modified T cells in a murine Ewing sarcoma xenograft model. **A** Schematic illustration of the experimental design. **B** Numbers of human CD3 + T cells in dissociated tumor xenografts by flow cytometry on day 11. Significance was assessed using ANOVA on Ranks. **C** Amounts of tTF-NGR or tTFmut-NGR in tumor lysates by ELISA on day 11. Significance was assessed using ANOVA on Ranks. **D** Tumor volumes on days 4, 7 and 11 after administration of CAR T cells. n = numbers of mice/cohort alive (of 5 per cohort) at the individual point in time. All analyses were started with 5 mice per cohort and either shown as individual data or as means. **E** Schematic illustration of the experimental design for in vivo efficacy. **F** Tumor volumes on the indicated times after administration of CAR T cells. n = numbers of mice/cohort alive at the individual point in time. **G** Overall survival by Kaplan-Meier analysis (left panel) and outcome analysis in the 3 cohorts (right panel). *P*-values show pair-wise Log-Rank test results. All analyses were done with 9 mice (GD2_tTF, GD2_tTFmut) or 8 mice (CD19_tTF) per cohort and shown as individual data or as means.

To recapitulate whether CAR T cells with inducible tTF-NGR infiltrate human EwS xenografts and release tTF-NGR into the TME, we s.c. injected EwS cells of the G_D2_-pos, CD19-neg EwS cell line A4573 into NSG mice (Fig. 5A). Murine myeloid cells have emerged as a major barrier to the antitumor activity of CAR T cells in EwS xenograft models, motivating the use of selective depletion strategies to enable efficacy of T cell immunotherapy in immunodeficient mice [31, 32]. On the basis of own pilot experiments (Suppl. Fig 3), we chose to use continuous treatment with 5-fluorouracil (5-FU) at 10 mg/kg/d [33] upon a tumor volume of 50 mm^3^ for myeloid cell depletion. To allow the tumors to establish a vascular architecture, in this first experiment we did not start T cell injections before larger tumors of 300 to 400 mm^3^ had formed. Two consecutive doses of T cells (5 × 10^6^/dose) were given by i.p. administration in a 3-day interval, along with i.p. IL-2. Again, T cells transduced to express GD2_tTFmut or CD19_tTF were used to treat control cohorts of mice. This time, the experiment was terminated on day 11. Flow cytometry analysis demonstrated significant proportions of infiltrating human T cells in mice treated with the G_D2_-specific CAR T cell products in contrast to T cells targeting the irrelevant antigen CD19 (Fig. 5B), although again with interindividual variability.

tTF-NGR was detected by ELISA in 3 of 5 tumors after treatment with GD2_tTF engineered T cells at different concentrations, and tTFmut-NGR was not found in any of the tumors (Fig. 5C). Tumor volumes at autopsy on days 7 and 11 were noticeably lower in the cohort of mice treated with GD2_tTF engineered T cells compared to both GD2_tTFmut or CD19_tTF, but the experiment was not designed and powered for this statistical analysis (Fig. 5D).

To investigate whether CAR T cells with inducible tTF-NGR have therapeutic activity, we then repeated the experiment, with some modifications (Fig. 5E). To ensure consistent reduction of myeloid suppressor cells in the TME, we now continued 5-FU treatment once every day. Second, to enable longer follow-up of the mice in this highly aggressive model, we consistently started T cell therapy at small tumor volumes of 100–150 mm^3^, and third, we administered a third dose of T cells again in a 3-day interval. Whereas at the experimental endpoint on day 40 after CAR T cell administration, all mice in the control cohort died of disease, G_D2_-specific CAR T cell therapy halted tumor growth in a proportion of animals. Of 9 mice receiving the therapeutic CAR T cell construct, GD2_tTF, 3 obtained and maintained complete remissions, compared to 1 out of 9 mice treated with GD2-specific CAR T cells releasing mutant tTF-NGR lacking coagulatory activity (Fig. 5F, G). Analysis of tumor growth curves revealed substantial growth inhibition in the mice treated with both G_D2_-specific CAR T cell treated cohorts, GD2_tTF and GD2_tTFmut (Fig. 5F). A noticeable additional delay of tumor growth was seen after administration of GD2_tTF transduced T cells compared to T cells secreting the mutant control construct without coagulatory activity. However, with the sample size estimation for the experimental groups this difference between GD2_tTF and GD2_tTFmut did not reach statistical significance, nor did the trend for a benefit on survival by Kaplan Meier analysis (Fig. 5G).

In conclusion, a consistent trend for superiority of GD2_tTF engineered T cells over both other groups was noticed across all parameters of response, tumor growth and survival.

### Conditions mimicking hypoxia enhance antigen-induced granzyme B secretion by CAR T cells

According to our original hypothesis, tTF-NGR secretion induced by CAR T cell engagement of target antigen would support effector T cell responses within the hostile TME of solid tumors by an immune-independent mechanism. However, at the times assayed, we failed to detect clear signs of locoregional vascular disruption by histology in our animal model (Fig. 4E). This argues against tumor cell death as a consequence of locoregional infarction, as consistently found with the systemic administration of tTF-NGR [6, 7, 34]. This could in part be explained by the low and variable concentrations of tTF-NGR released in our model (Figs. 4C, 5C), which may have been insufficient to induce irreversible clotting of tumor blood vessels.

**Fig. 6:**
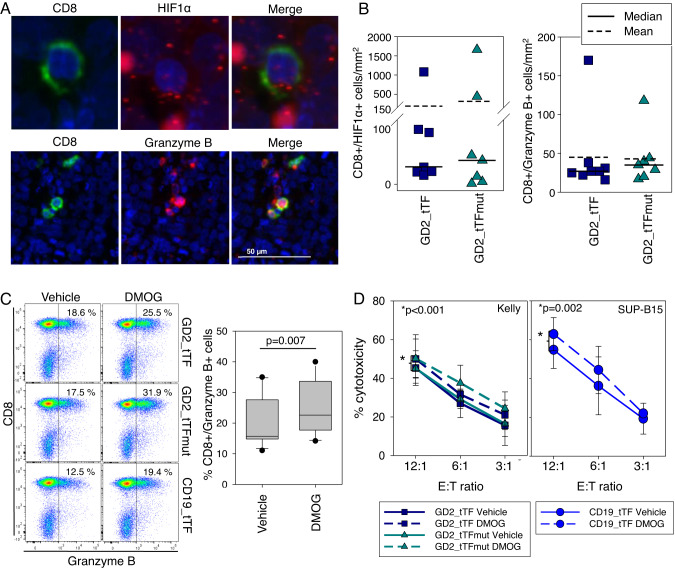
Analysis of hypoxia as a mechanism for tTF-NGR. **A** Example of co-staining of CD8 (immunofluorescence), HIF1α (RNAish) and DAPI (upper panel), and of CD8, granzyme B and DAPI (lower panel, immunofluorescence) on paraffin-embedded tumor tissues. **B** Quantification analysis using Inform Software and R script from 3-4 xenograft tissues (7 days after CAR T cell injection) and 5 xenograft tissues (11 days after CAR T cell injection) of the above staining. **C** Granzyme B expression on T cells transduced with the indicated constructs cultured in the presence or absence of the hypoxia-mimetic DMOG (50 µM) or DMSO (control), respectively, for 72 h. Shown is one example of 4 donors and a summary of all 4 donors and all three constructs. Significance was measured using the paired t-test. **D** Cytolysis of G_D2_-positive neuroblastoma cells (Kelly, left panel) and CD19-positive cells (SUP-B15, right panel) by CAR T cells with the indicated G_D2_- or CD19-specific CAR constructs coexpressing either tTF-NGR or tTFmut-NGR as control, respectively, after culture in the presence or absence of the hypoxia-mimetic DMOG (50 µM) or DMSO as control for 72 h. Shown is the mean with standard deviation of 4 individual donors. Significance was measured using a paired t-test over all E:T ratios.

More subtle or transient effects could potentially explain the effects of GD2_tTF-engineered T cells on tumor parameters that we observed. Various reports suggest that mild hypoxia can enhance the cytotoxic effector responses of human T cells [35, 36]. To evaluate potential effects of tTF-NGR on the expression of granzyme B and/or on hypoxia-inducible factor (HIF)-1α expression on CD8 + CAR T cells infiltrating tumor xenografts, we costained xenografts obtained at autopsy from the above in vivo experiments with antibodies against human CD8 and granzyme B or used RNAish for detection of HIF-1α, an early marker for hypoxia, along with DAPI. Neither HIF-1α nor granzyme B (Fig. 6A, B) expression differed between tumors from mice treated with GD2_tTF-NGR or the control. This analysis at a single and late point in time may have missed earlier events. Thus, to evaluate potential effects of hypoxia on CAR T cell effector functions in vitro, we mimicked hypoxic conditions by adding the prolyl-hydroxylase inhibitor dimethyloxalylglycine (DMOG), a competitive inhibitor of HIF-hydroxylated prolyl hydroxylase and thereby hypoxia-mimetic agent, to CAR T cell cultures. T cells transduced with either of the experimental or control constructs significantly upregulated granzyme B in the presence of DMOG (Fig. 6C). This effect was observed for all 3 CAR T cell populations as expected. Further, cytotoxicity assays using DMOG-pretreated or untreated CAR T cells as effector cells demonstrated significantly increased target cytolysis (Fig. 6D), again for all three constructs as expected, upon coculture with target cells expressing the respective antigen. Thus, induction of the cytolytic machinery in response to even mild or transient hypoxia caused by secretion of functional tTF-NGR in the in vivo experiments may have contributed to the antitumor activity of CAR T cells.

## Discussion

CAR T cell micropharmacies engineered to secrete antigen-inducible tTF-NGR exerted antitumor activity against EwS xenografts. Superiority to a truncated variant lacking coagulatory activity was noticeable and consistent, though the difference in effect size was not strong enough to show statistical significance with the numbers of animals present in the cohorts. Concerning the mode of action, we could not confirm our initial hypothesis that delivery of tTF-NGR to blood vessels in the TME would induce thrombosis with subsequent tumor infarction and regression. That is despite the fact that we established all major prerequisites for the anticipated mode of action: In response to antigen exposure, the engineered human T cells produce and secrete recombinant tTF-NGR. The fusion protein released by T cells efficiently mimics the capacity of TF to activate the extrinsic coagulation cascade, as reported for tTF protein recombinantly expressed in *E. coli* [6, 7], which structurally differs by its glycosylation pattern. Release of tTF-NGR strictly relied on tumor antigen-induced T cell activation, minimizing the risk of uncontrolled systemic pro-coagulatory activity. Coengineering with tTF-NGR did not affect the activation and effector responses of the T cells.

Pharmacokinetic differences could explain why tTF-NGR secreted by CAR T cells into the tumors failed to induce microscopically visible vascular clotting. The amounts of tTF-NGR detected in the tumors were comparatively low. According to our pharmacokinetic data in mouse tTF-NGR studies and in the human phase I trial, such concentrations are probably insufficient to exert the strong effect seen with systemic administration of the fusion protein. Blood peak concentrations (C_max_) of >700 ng/mL (10 min after push injection) were found for therapeutically active schedules inducing tumor vascular occlusion and tumor infarction in mouse xenograft models [37]. In the human phase I study, blood C_max_ values for tTF-NGR of 140 ng/mL upon application of 1 mg/m^2^ and 600 ng/mL upon 3 mg/m^2^ body surface area were measured with these doses leading to selective inhibition of tumor blood flow [8]. In the experiments presented here, not all tumors contained measurable quantities by ELISA, and the concentrations were around 80 pg/mL tumor tissue, with considerable variability among individual tumors. Although these findings must be interpreted with caution, since the late timepoint at which we analyzed the tumors may not have adequately represented earlier events during the course of the CAR T cell response, the concentrations observed were several orders of magnitude lower than those found upon application of active tTF-NGR doses in mice and humans leading to vascular occlusion. One limitation of the expression of an inducible transgene under NFAT promoter control is that this system does not allow to regulate or enhance the amounts of protein released. While the vector design was adopted from a previous successful approach to engineer T cells for secretion of IL-12 [10], higher amounts of protein are potentially needed to exert the intended mechanism. From the retroviral vector used in this report, we have now switched to a lentiviral one-vector expression system, with higher efficiency of transgene expression [23]. Besides, additional novel technologies for this purpose are being developed [38]. Moreover, our inducible system relies on antigen-induced activation and proliferation of CAR T cells. Despite our attempts to eliminate myeloid suppressor cell populations that impede the function of CAR T cells [32] using 5-FU [33], the antitumor activity of both standard and tTF-NGR-secreting CAR T cells was limited. The kinetics of CAR T cell activation and expansion following engagement by G_D2_ on tumor cells in this hostile TME may have been inadequate to induce the concerted release of the agent. This consideration is well in line with the limited numbers of CAR T cells we detected in the tumors at the time of autopsy. Constitutive rather than inducible expression of the transgene may be one way to ensure release of higher concentrations of the agent, but may compromise the safety of the strategy by limiting its selectivity.

Finally, our strategy requires that tTF-NGR, released by CAR T cells in response to tumor cells embedded into perivascular stroma, will reach the endothelial side of the vascular tumor bed. We hypothesize that G_D2_-specific CARs can achieve this purpose with the following arguments: First, the endothelial lining in tumor vessels is known to be permeable [39, 40], allowing protein secreted by CAR T cells into the perivascular tumor stroma to penetrate into the luminal side of the vessels. Second, tumor neovessels are often lined by tumor cells as a consequence of tumor cell vasculogenic mimicry [40]. Specifically, Ackermann et al. reported that CD99-expressing tumor cells contribute to endothelial cell formation in a xenograft model of EwS [41], which implies that G_D2_-specific CAR T cells are highly likely to interact with tumor cells (and release tTF-NGR) also on the luminal side of neovessels, the main location of the pro-coagulatory activity of tTF-NGR.

Even though we failed to prove our initial mechanistic hypothesis, consistent evidence supports an additive effect of tTF-NGR release by CAR T cells against solid tumors in our in vivo models. Based on previous reports that mild hypoxia, e.g. induced by blockade of vascular endothelial growth factor A, augments the activation and effector phenotype and functions of CD8 + T cells by initiating the HIF1α program [36, 42, 43], and on our own new finding that a hypoxia-mimetic agent significantly enhances the cytolytic capacity of gene-engineered CAR T cells, we have reason to hypothesize that procoagulatory effects of even small amounts of tTF-NGR in tumor vessels may create mild hypoxic conditions for superior CAR T cell activity within the TME.

Overall, this study provides further proof of the concept of using CAR T cells as micropharmacies to deliver a protein into the tumor microenvironment. The strategy was first developed with the aim to provide cytokine support, e.g. by IL-12 and later IL-15 or IL-18 [10, 44–50], then extended to various alternative payloads designed to improve the potency of receptor-engineered T cells [51–53]. Recently, Gardner et al. have proposed to engineer CAR T cells to secrete an enzyme that activates a systemically administered small-molecule prodrug, which allowed significant lysis also of antigen-negative tumor cells in mouse xenograft models [38]. Our approach to co-target the vascular microenvironment by a one-vector engineering strategy is a novel concept to the best of our knowledge. Whereas most previous investigators have used constitutive expression vectors, allowing for some tumor enrichment of the secreted payload by antigen-induced CAR T cell proliferation, we favor an inducible expression mode that ensures locoregional and time restriction of any potential effect of the transgene which cedes after elimination of all antigen-positive targets, since it provides an additional layer of safety and will allow to use CAR T cells as vehicles also for agents with a more limited therapeutic window.

In summary, next generation immunotherapies relying on the use of CAR T cell micropharmacies deserve further development. Technical improvements, such as more effective vector technologies and integrated solutions for higher CAR T cell infiltration and expansion in the TME of solid tumors with subsequent higher amounts of a secreted payload into the tumor should be pursued to overcome current limitations.

### Supplementary information

Suppl Figures

## Data Availability

Additional data beyond the data found in the article are available from the corresponding author on reasonable request.
