# Supplementary material for: CAR T cells as micropharmacies against solid cancers: Combining effector T-cell mediated cell death with vascular targeting in a one-step engineering process
Source: Cancer Gene Ther. 2023 Jun 30;30(10):1355–68. doi: 10.1038/s41417-023-00642-x (PMC10581901; doi:10.1038/s41417-023-00642-x)
Supplement: Supplementary file 1 — Suppl Figures [file 41417_2023_642_MOESM1_ESM.pdf]

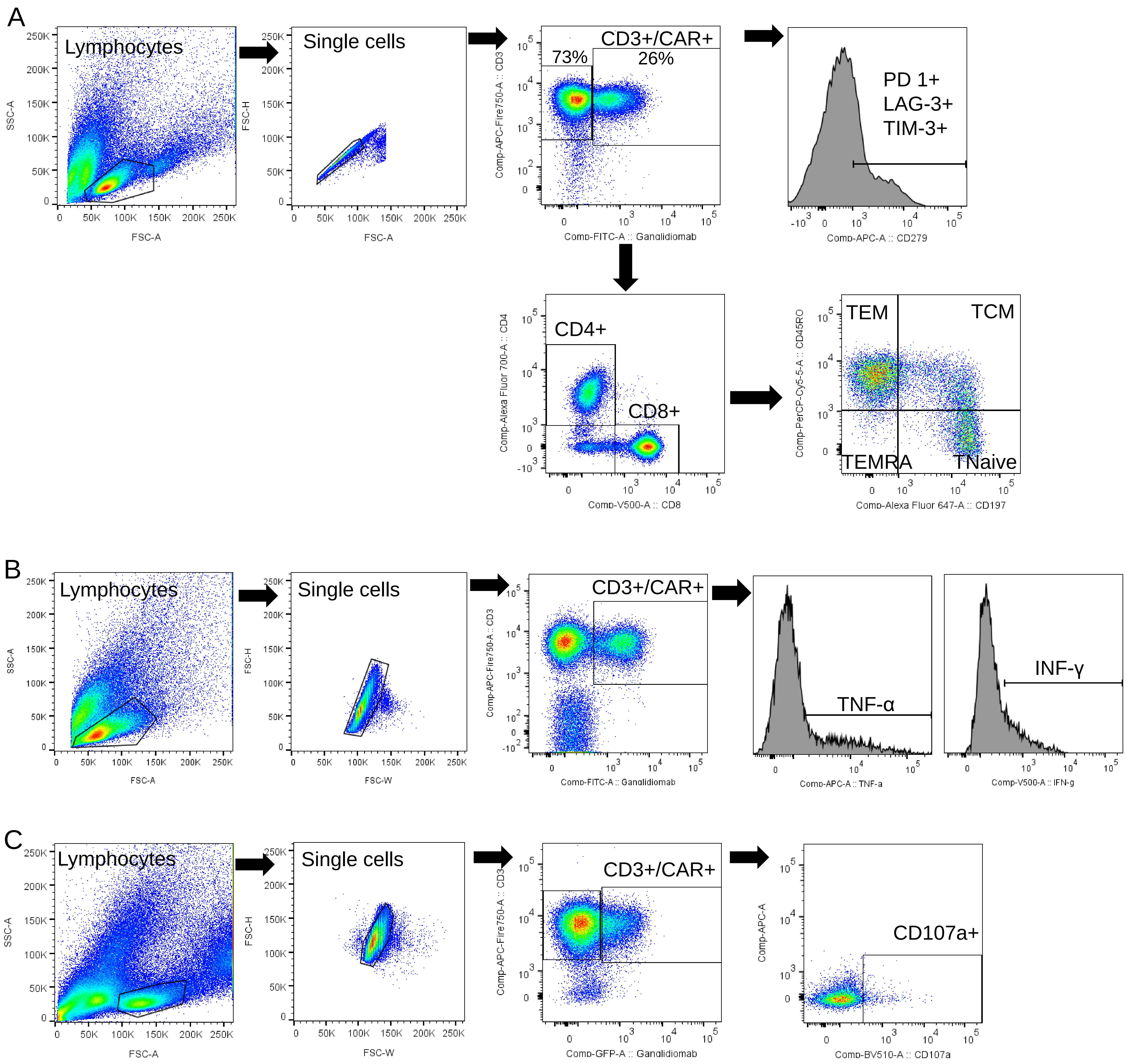

Supplementary Figure 1. Gating strategy for the flow cytometry analysis in Figure 2. (A) Analysis of surface expression of exhaustion markers PD-1, LAG-3 and TIM-3 on transduced T cells, CD4 and CD8 ratios and proportions of CD3+ T cells with naive (TNaive), central memory (TCM), effector memory (TEM) and CD45RA+ effector memory (TEMRA) phenotypes, all assessed on day 14 after transduction by flow cytometry. (B) Intracellular staining for TNF-α and IFN-γ upregulation in CD3+/CAR+ T cells after stimulation with tumor cells. (C) CD107a upregulation on CD3+/CAR+ T cells in response to coincubation with tumor cells. Plots are from one representative sample.

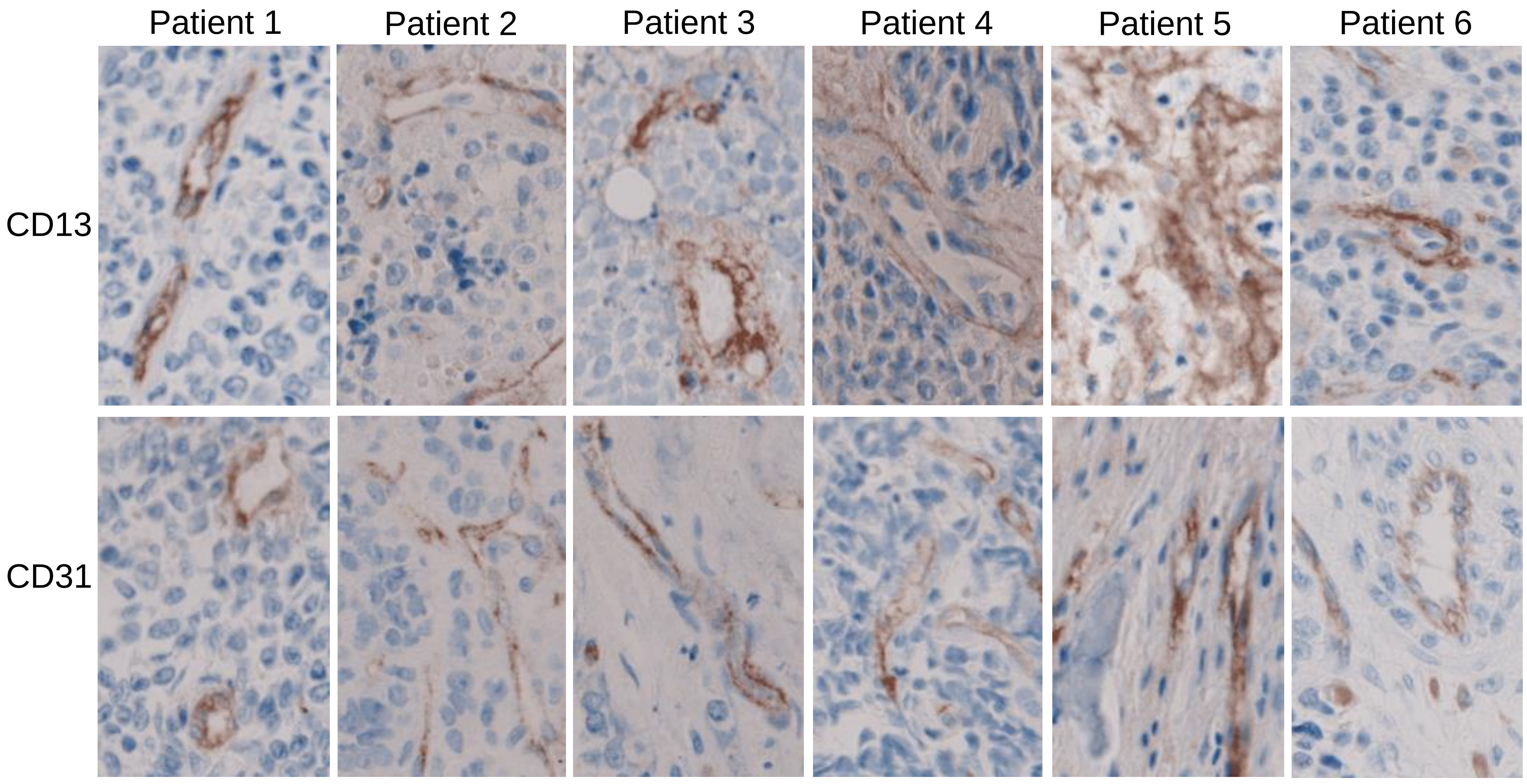

Supplementary Figure 2. Analysis of CD13 and CD31 expression on paraffin-embedded tissues of EwS patient biopsies by immunohistochemistry. The upper panel shows the staining with the anti-human CD13 antibody (clone SP10087, Cell Marque, USA) and the lower panel the staining with the CD31 antibody (clone JC70, Cell Marque, USA). All six individual patients analyzed were positive for both CD13 and CD31.

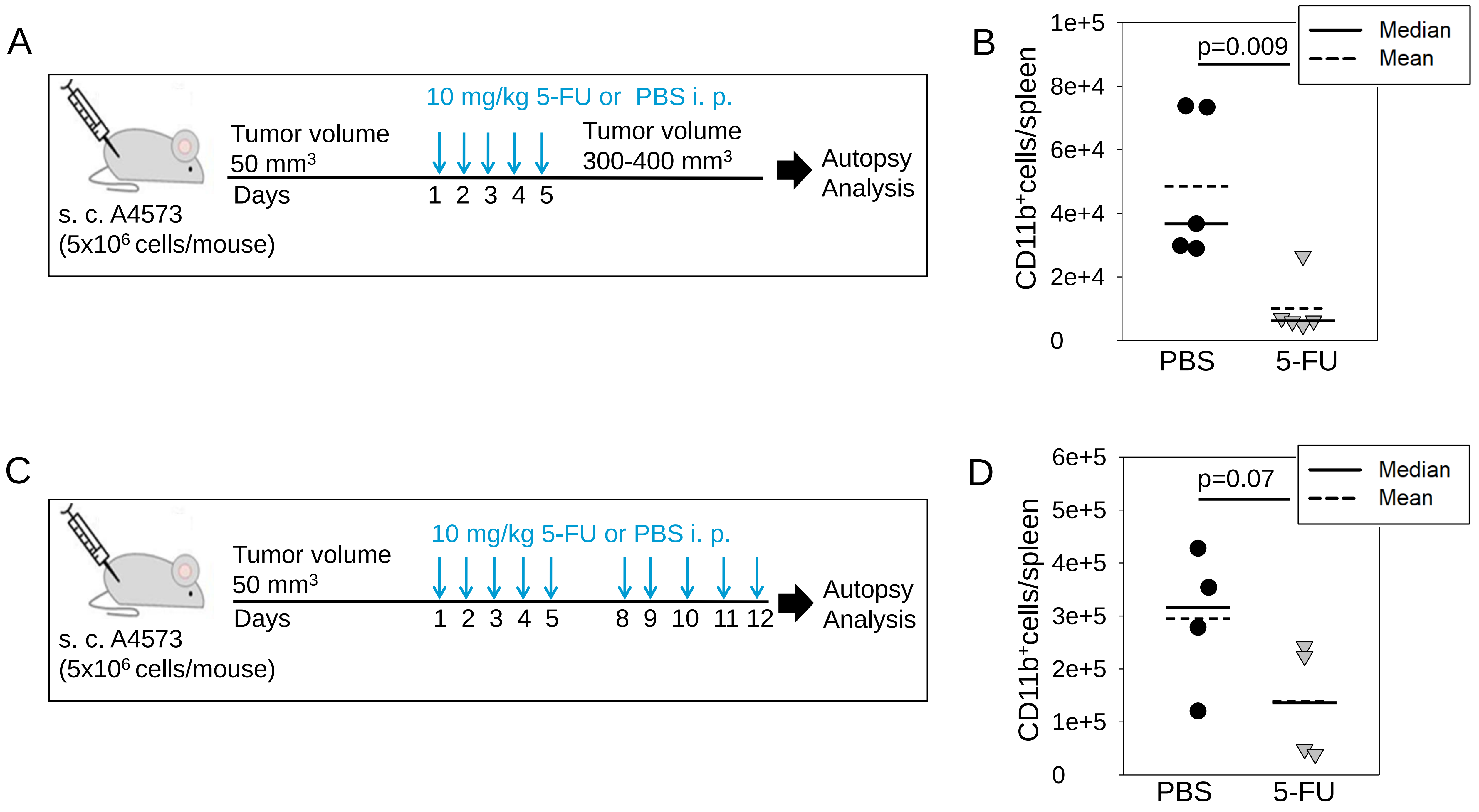

Supplementary Figure 3. Reduction of CD11b+ murine myeloid cells by pretreatment with 5-FU. (A) Schematic illustration of the experimental design: Ten mice were subcutaneously injected with $5x10^6$ A4573 EwS cells each. Upon a tumor volume of 50 mm^3, therapy was started with intraperitoneal injection of 10 mg/kg 5-FU or PBS as a control into five mice each and on five consecutive days. The mice were sacrificed after reaching tumor volumes of 300 to 400 mm^3 and numbers of CD11b+ cells were quantified in spleens by flow cytometry (B). (C) Schematic illustration of the experimental design: Eight mice were subcutaneously injected with $5x10^6$ A4573 EwS cells each. Upon a tumor volume of 50 mm^3, therapy was started with intraperitoneal injection of 10 mg/kg 5-FU or PBS as a control into four mice each on 5 consecutive days over two weeks. The mice were sacrificed on day 13 and numbers of CD11b+ cells were quantified in spleens by flow cytometry (D). Significance was assessed by using t-test.
